# Publication of nuclear magnetic resonance experimental data with semantic web technology and the application thereof to biomedical research of proteins

**DOI:** 10.1186/s13326-016-0057-1

**Published:** 2016-05-05

**Authors:** Masashi Yokochi, Naohiro Kobayashi, Eldon L. Ulrich, Akira R. Kinjo, Takeshi Iwata, Yannis E. Ioannidis, Miron Livny, John L. Markley, Haruki Nakamura, Chojiro Kojima, Toshimichi Fujiwara

**Affiliations:** 1 Institute for Protein Research, Osaka University, 3-2 Yamadaoka, Suita, Osaka 565-0871 Japan; 2 Department of Biochemistry, University of Wisconsin-Madison, Madison, WI 53706 USA; 3 Department of Informatics & Telecommunications, University of Athens, Athens, Greece; 4 Department of Computer Sciences, University of Wisconsin-Madison, Madison, WI 53706 USA

**Keywords:** NMR, BMRB, Database, XML, RDF

## Abstract

**Background:**

The nuclear magnetic resonance (NMR) spectroscopic data for biological macromolecules archived at the BioMagResBank (BMRB) provide a rich resource of biophysical information at atomic resolution. The NMR data archived in NMR-STAR ASCII format have been implemented in a relational database. However, it is still fairly difficult for users to retrieve data from the NMR-STAR files or the relational database in association with data from other biological databases.

**Findings:**

To enhance the interoperability of the BMRB database, we present a full conversion of BMRB entries to two standard structured data formats, XML and RDF, as common open representations of the NMR-STAR data. Moreover, a SPARQL endpoint has been deployed. The described case study demonstrates that a simple query of the SPARQL endpoints of the BMRB, UniProt, and Online Mendelian Inheritance in Man (OMIM), can be used in NMR and structure-based analysis of proteins combined with information of single nucleotide polymorphisms (SNPs) and their phenotypes.

**Conclusions:**

We have developed BMRB/XML and BMRB/RDF and demonstrate their use in performing a federated SPARQL query linking the BMRB to other databases through standard semantic web technologies. This will facilitate data exchange across diverse information resources.

**Electronic supplementary material:**

The online version of this article (doi:10.1186/s13326-016-0057-1) contains supplementary material, which is available to authorized users.

## Findings

### Background

**Fig. 1:**
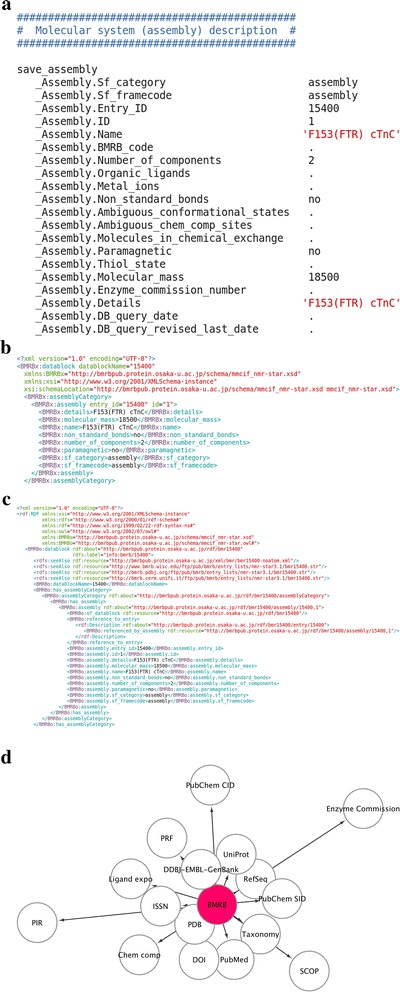
The sequential data conversion of a BMRB entry from NMR-STAR format. **a** Part of a BMRB entry in NMR-STAR format. Parts of the entry have been converted to **b** XML format and **c** RDF format. **d** Schematic representation of linked external information resources, where shorter distances from the BMRB represent closer relationships with the BMRB

The BioMagResBank (BMRB; http://www.bmrb.wisc.edu/) is a worldwide data repository for experimental and derived data gathered from nuclear magnetic resonance (NMR) spectroscopic studies of biological molecules [1]. For more than 15 years, the BMRB has used and developed the NMR-STAR format based on the STAR format specifications [2–4] for data archiving and data exchange (see Fig. 1a). The NMR-STAR Dictionary, acting as ontology for NMR-STAR data, continues to be improved and expanded to keep up with the needs of the biomolecular NMR community. The most important parameter archived in the BMRB is assigned chemical shifts, which can be used directly to determine protein secondary structure and to assist in the determination of their solution structures, to identify interactions of small molecules with target proteins for drug discovery, and to characterize protein-protein interactions.

As of 2015, the BMRB archive contains more than 10,000 entries, and ~800 new entries are being added each year. This growing biological data archive has raised researchers’ interest in integrating the diverse biological information resources to form new hypotheses for understanding biological functions and phenomena. The best approach for enhancing interoperability of the BMRB would be to convert the archive into standard web formats, XML and RDF, using a data structure that corresponds closely to the NMR-STAR ontology described by an XML schema and OWL.

## Methods

We have extended the NMR-STAR Dictionary to accommodate the derived data repositories on BMRB, such as LACS validation reports [5], structural annotations using PACSY [6] and Protein Blocks [7], etc., followed by translation of the dictionary to an XML schema [8] (BMRB/XML Schema), using the PDBx/mmCIF Dictionary Suite developed by the Research Collaboratory for Structural Bioinformatics (RCSB) Protein Data Bank (PDB) (http://sw-tools.rcsb.org/). To automate the XML conversion of BMRB entries, we have developed a software suite (the BMRBxTool) that generates XML documents and validates their format and data consistency according to the BMRB/XML schema. EXtensible Stylesheet Language (XSL) transformation [9] was applied to generate BMRB entries in RDF format from the corresponding XML documents. Along with the RDF, we also provide its ontology written in RDF/RDFS/OWL syntax as BMRB/OWL [10, 11], which is a direct translation of the BMRB/XML schema. To bridge different data models between the XML tree and the RDF directed graph, we have introduced abstract OWL classes and RDF properties to the ontology [12]. For the data conversion from XML to RDF in accordance with the principles of Linked Data [13] and recommendations widely accepted by biological database community [14], we have developed a software suite called BMRBoTool. We have tested as many as thirty SPARQL queries to show how NMR experimental data can be retrieved. In a case study to demonstrate a federated SPARQL query, we performed a search for phenotypes annotated with information for SNPs from the human genome by integrating three SPARQL endpoints: the BMRB, UniProt, and OMIM [see Additional file 1 for details].

## Results and discussion

On our portal site (http://bmrbpub.protein.osaka-u.ac.jp/, hereafter abbreviated as ‘~/’), we have archived the BMRB/XML (~/archive/xml/), as shown in Fig. 1b. The BMRB/RDF derived from the reduced version of the BMRB/XML (lacking bulky information such as atomic coordinates and NMR restraints) has also been archived (~/archive/rdf/) as shown in Fig. 1c. A bulk download service is available using the *rsync* protocol that helps users mirror the latest data collections, which are updated periodically. A schematic RDF graph of linked databases is illustrated in Fig. 1d. Owing to the machine readability of the XML format, the BMRB/XML provides users with an excellent starting point to develop new tools for use in biochemistry and structural biology. The BMRB/RDF and associated web services enable the integration of the BMRB archive with other biological databases, which may facilitate flexible data exchange and knowledge discovery.

**Fig. 2:**
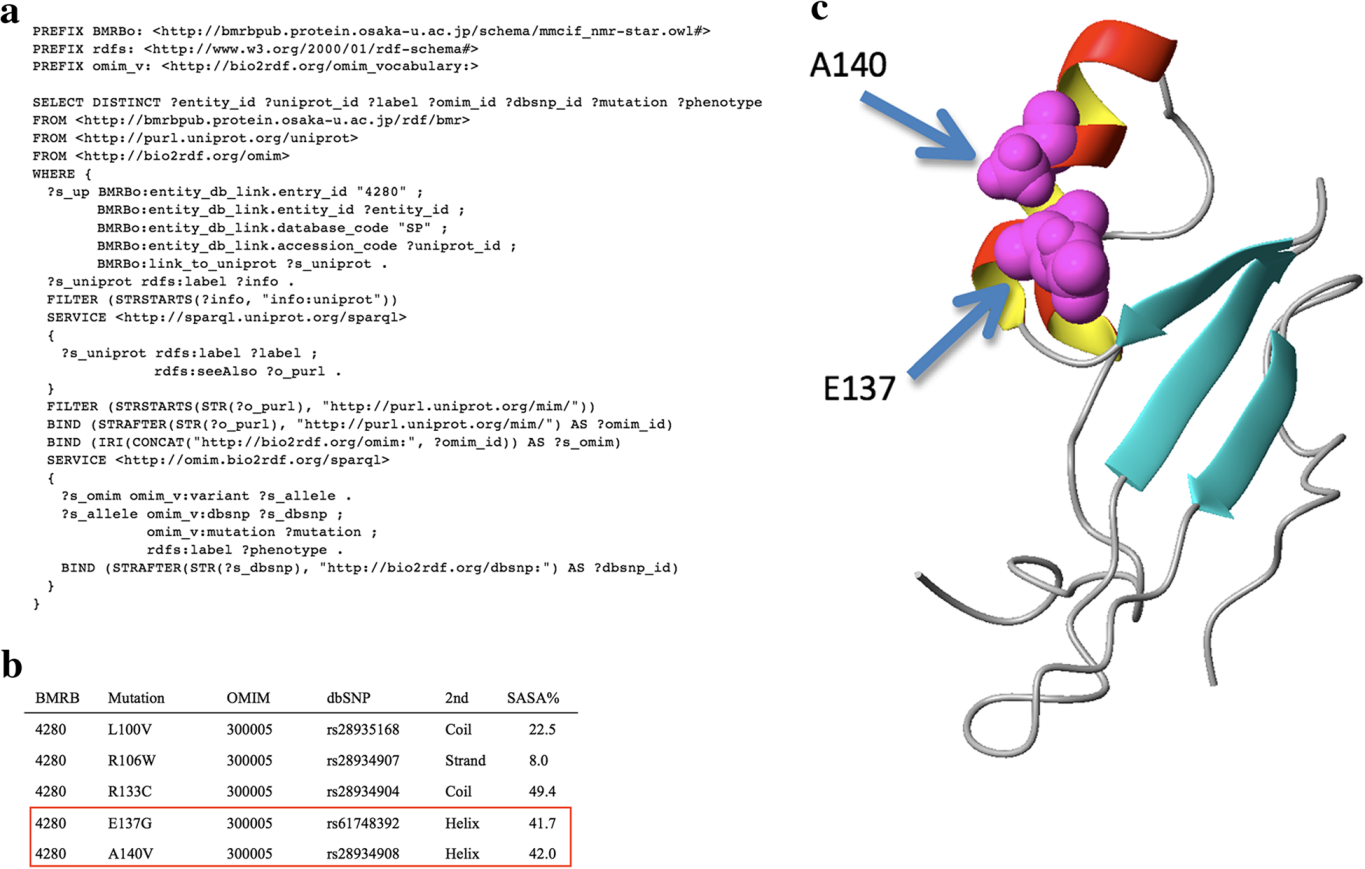
Federated search using multiple SPARQL endpoints of the BMRB, UniProt, and OMIM. **a** An example of a SPARQL query begins from the BMRB entry. **b** Results performed by the SPARQL query for BMRB entry 4280, showing the BMRB ID, mutation, OMIM ID, dbSNP ID, secondary structure, and SASA. **c** Ribbon models of NMR structure (PDB: 1QK9) of MECP2. The side-chains (space-filling model) for the mutation (E137 and A140) exposed to solvent are responsible for X-linked mental retardation [18]

Furthermore, we provide a SPARQL endpoint for querying the BMRB/RDF (~/search/rdf) in the same way as Bio2RDF [15] does. The RDF query language, SPARQL [16], realizes data exchange between databases in a concise syntax. The key feature of SPARQL is its capability of joining remote SPARQL endpoints in what is called a federated SPARQL query [17]. We confirmed the feasibility of such a query (see Fig. 2a), by demonstrating search and classification of SNPs in an associated BMRB entry. In the case study, we successfully collected residues in a BMRB entry whose sequences correspond to SNPs annotated by OMIM, as shown in Fig. 2b. The search results (Fig. 2c) were represented by structural parameters archived in the BMRB [see also Additional file 1 for methods applied and all results, chapter 3], allowing users to infer biological relationships between the phenotype annotated SNPs and structural features in human proteins (see Fig. 2c). The results show that the BMRB/RDF offers a promising approach for integrating biophysical information derived from biological NMR spectroscopy with other bioinformatics resources of interest in biological and medical science research.

## Additional file

Additional file 1: Further detail of the BMRB/XML, BMRB/RDF and web services including SPARQL endpoint. Complete results of the SPARQL query (Fig. 2a) and many other SPARQL query examples are also available. (PDF 6814 kb)

## Abbreviations

BMRB: BioMagResBank
MECP2: methyl-CpG-binding protein 2
NMR: nuclear magnetic resonance
OMIM: Online Mendelian Inheritance in Man
OWL: web ontology language
RDF: resource description framework
RDFS: RDF schema
SASA: solvent accessible surface area
SNP: single nucleotide polymorphism
SPARQL: SPARQL Protocol and RDF Query Language
XML: eXtensible Markup Language
