# Supplementary material for: Publication of nuclear magnetic resonance experimental data with semantic web technology and the application thereof to biomedical research of proteins
Source: J Biomed Semantics. 2016 May 5;7:16. doi: 10.1186/s13326-016-0057-1 (PMC5143449; doi:10.1186/s13326-016-0057-1)
Supplement: Additional file 1: — Further detail of the BMRB/XML, BMRB/RDF and web services including SPARQL endpoint. Complete results of the SPARQL query (Fig. 2a) and many other SPARQL query examples are also available. (PDF 6814 kb) [file 13326_2016_57_MOESM1_ESM.pdf]

Publication of nuclear magnetic resonance experimental data with semantic web technology and the application thereof to biomedical research of proteins

Supporting Data

Masashi Yokochi[1], Naohiro Kobayashi[1], Eldon L. Ulrich[2], Akira R. Kinjo[1], Takeshi Iwata[1], Yannis E. Ioannidis[3], Miron Livny[4], John L. Markley[2], Haruki Nakamura[1], Chojiro Kojima[1], Toshimichi Fujiwara[1,*]

[1]*Institute for Protein Research, Osaka University, 3-2 Yamadaoka, Suita, Osaka, 565-0871, Japan*

[2]*Department of Biochemistry, University of Wisconsin-Madison, Madison, WI 53706, USA*

[3]*Department of Informatics & Telecommunications, University of Athens, Athens, Greece*

[4]*Department of Computer Sciences, University of Wisconsin-Madison, Madison, WI 53706, USA*

* To whom correspondence should be addressed.

Tel: +81 6 6879 8597

Fax: +81 6 6879 8599

Email: tfjwr@protein.osaka-u.ac.jp

Contents

1 BMRB/XML

1.1 BMRB/XML Schema 3

1.2 Integration of data repositories on BMRB into XML format 7

1.3 BMRB/XML data files 10

1.4 XML schema validation and data remediation 10

1.4.1 Null data for mandatory entry fields 11

1.4.2 Violations in enumerators 12

1.4.3 Remediation of database accession codes 12

1.4.4 Statistics of data remediation by BMRBxTool 14

2 BMRB/RDF

2.1 BMRB/OWL 15

2.1.1 Translation protocol from XML Schema to OWL ontology 15

2.1.2 Comparison with other translation tool 20

2.2 BMRB/RDF data files 20

2.2.1 Translation protocol compliant with principles of Linked Data 20

2.2.2 Statistics on BMRB/RDF 23

3 Data access

3.1 Basic look-up service 27

3.2 SPARQL based query service (SPARQL endpoint) 29

3.3 Federated SPARQL query 29

3.3.1 Application to data exchange (Comparative survey of trends in publications between BMRB and PDB) 29

3.3.2 Application to knowledge discovery (Search and classification of SNPs in associated BMRB entities) 33

3.3.3 Summary of SPARQL queries using BMRB/RDF 40

REFERENCES 42

1. BMRB/XML

1.1 BMRB/XML Schema

The NMR-STAR Dictionary and PDB Exchange Dictionary are ontologies of NMR-STAR data and PDBx/mmCIF data, respectively. The both are derived from STAR/DDL compliant dictionary [1-3], and use the same STAR syntaxes and an architecture in which a single 'datablock' as defined by the dictionary constitutes a collection of categories. The PDBx/mmCIF Dictionary Suite developed by RCSB PDB (http://sw-tools.rcsb.org/), has been used to generate an XML Schema for the BMRB/XML (BMRB/XML Schema) [4-5] from the NMR-STAR Dictionary in a way comparable to that of generating the PDBML Schema from the PDB Exchange Dictionary [6]. Therefore, both architectures of the two XML Schemas are equivalent. The correspondences between metadata in the two dictionaries and XML Schema elements are summarized in Table S1. The prefix of the XML namespace for the BMRB/XML Schema is 'BMRBx', while 'PDBx' is used for the PDBML Schema. The XML schema is available at ~/schema/mmcif_nmr-star.xsd, hereafter '~/' stands for http://bmrbpub.protein.osaka-u.ac.jp/. Besides use of the fully automated translation, we have embedded direct links to the NMR-STAR Dictionary reference service (http://www.bmrb.wisc.edu/dictionary/) in the BMRB/XML Schema file (Figure S2). As a result, total 415 categories and 5090 data items are mapped to

schema objects preserving the canonical ontology so that the users familiar with NMR-STAR format can handle XML contents in a straightforward manner.

Symbolic representation in NMR-STAR format starts with a '$' character as defined by the STAR specification denote 'saveframe' pointers. However, the unique syntax has been avoided and replaced by the original name during XML conversion because the main purpose of generating XML documents is to be read by machine, in which a saveframe is addressed by an ID number rather than its name.

Table S1. Summary of correspondences between metadata of NMR-STAR Dictionary, PDB Exchange Dictionary and their XML Schemata

NMR-STAR Dic.	PDB Exchange Dic.	XML schema elements written in XPath syntax [7][a]
mmcif_nmr-star.dic		/xsd:schema[@xmlns:BMRBx='http://bmrbpub.protein.osaka-u.ac.jp/schema/mmcif_nmr-star.xsd']
	mmcif_pdbx.dic	/xsd:schema[@xmlns:PDBx='http://pdbml.pdb.org/schema/pdbx-v40.xsd']
Datablock	Datablock	/xsd:schema/xsd:complexType[@name='datablockType']
Datablock name	Datablock name	/Datablock/xsd:attribute[@name='datablockName']
Category group list	Category group list	Not mapped.
Category groups	Category groups	Not mapped.
Datablock-categories	Datablock-categories	/Datablock/xsd:all/xsd:element[@name='*category_name*Category'][@type='BMRBx:*category_name*Type' or @type='PDBx:*category_name*Type']
Parent-child	Parent-child	/xsd:schema/xsd:element[@name='datablock'][@type='BMRBx:datablockType' or @type='PDBx:datablockType']/xsd:key[@name='*key_name*']
Parent-child	Parent-child	/xsd:schema/xsd:element[@name='datablock'][@type='BMRBx:datablockType' or @type='PDBx:datablockType']/xsd:keyref[@name='*keyref_name*'][@refer='*key_name*']
Categories	Categories	/xsd:schema/xsd:complexType[@name='*category_name*Type']
Description	Description	/Category/xsd:annotation/xsd:documentation/text()
Primary keys	Primary keys	/Category/xsd:sequence/xsd:element[@name='*category_name*']/xsd:complexType/xsd:attribute[@name='*key*'][@use='required'][@type='xsd:string']
Items	Items	/Category/xsd:sequence/xsd:element[@name='*category_name*']/xsd:complexType/xsd:all/xsd:element[@name='*item_name*'][@minOcuurs='0'][@maxOccurs='1']
Description	Description	/Item/xsd:annotation/xsd:documentation/text()
Mandatory code	Mandatory code	/Item/[@minOccurs='1'][@maxOccurs='1']
Data types	Data types	/Item/[@type='xsd:string' or @type='xsd:integer' or @type='xsd:decimal']
Enumeration	Enumeration	/Item/xsd:simpleType/xsd:restriction[@base='xsd:*data_type*']/xsd:enumeration[@value='*enum_value*']
Unit types	Unit types	Defined as a set of enumerations as unit of measurement for value of corresponding data items.
Not used.	Sub categories	Not mapped.
Not used.	Matrix components	As it is.

[a]'/Datablock' , '/Category' and '/Item' indicate absolute location paths to metadata of the corresponding dictionaries; datablock, category and data item, respectively. The cyan colored schema elements highlight an important part of the context. The strings in italic font represent the symbols used in the metadata as nouns.

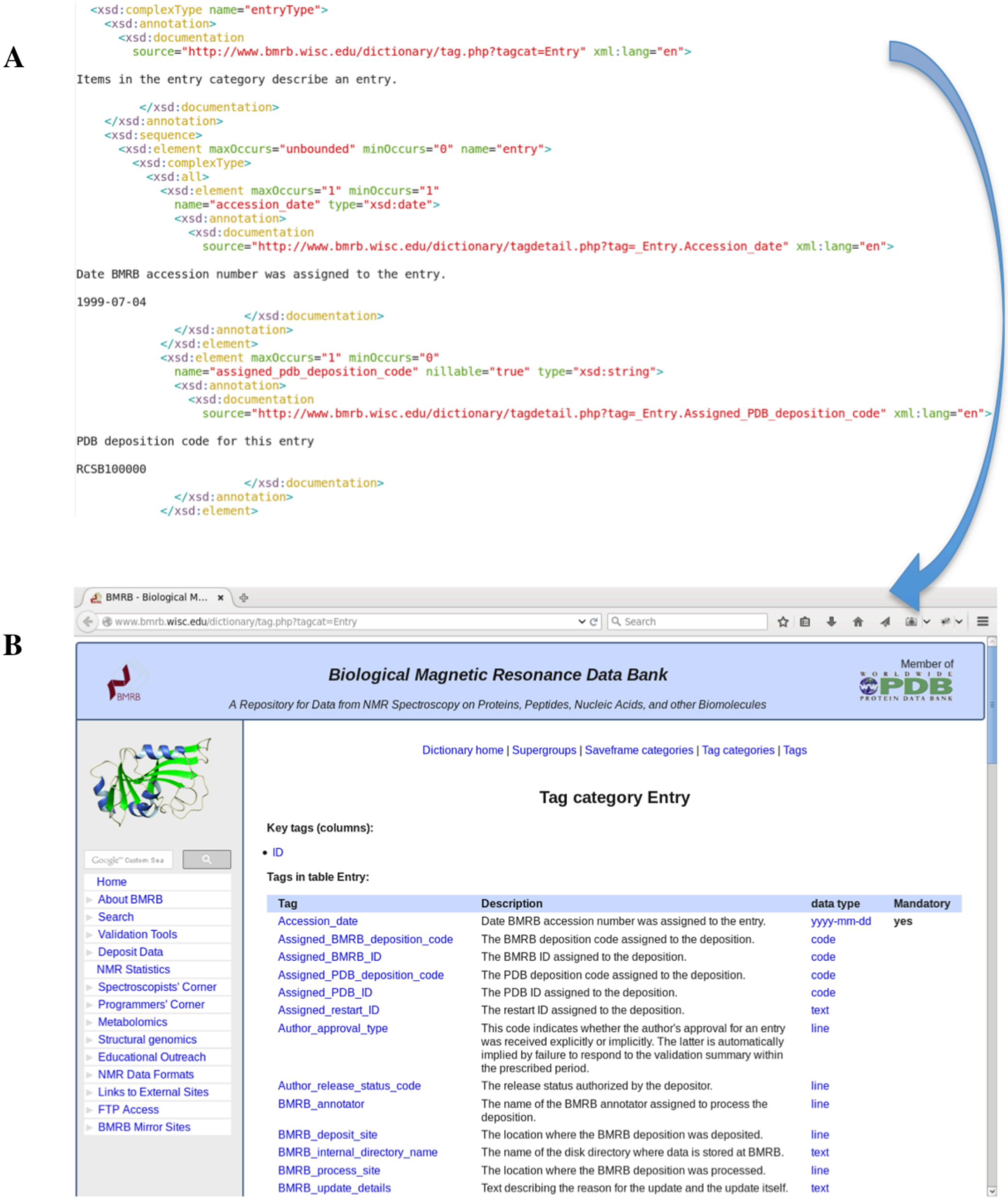

Figure S2. (**A**) BMRB/XML Schema file example of *entryType* schema object corresponding to *entry* category. Each schema object has original annotation in the NMR-STAR Dictionary together with a link to NMR-STAR Dictionary reference service that points corresponding categories and data items. (**B**) A web interface of the NMR-STAR Dictionary reference service that shows data items in the corresponding *entry* category.

1.2 Integration of data repositories on BMRB into XML format

BMRB maintains the following four data repositories: (i) quantitative NMR spectral parameters (e.g. assigned chemical shifts, J-coupling constants) and derived data (e.g. relaxation parameters, kinetics parameters), (ii) NMR restraints used for structure determination, (iii) time-domain spectral data and (iv) a NMR spectral database of metabolites and natural products. As of now, these NMR-STAR data have been distributed as separated files in different formats. For example, the NMR-STAR file consisting of data (i), has been available as two formats: current v3.1 (http://bmrb.pdbj.org/ftp/pub/bmrb/entry_lists/nmr-star3.1/) and the legacy v2.1 (http://bmrb.pdbj.org/ftp/pub/bmrb/entry_lists/nmr-star2.1/). Atomic coordinates, NMR restraints and experimental details relevant to NMR structure determination, which consist of (ii), are available as 'BMRB+PDB' data archive (http://bmrb.pdbj.org/ftp/pub/bmrb/nmr_pdb_integrated_data/coordinates_restraints_chemshifts/bmrb_plus_pdb/). The BMRB Metabolomics database, (iv), is accessible as web service (http://www.bmrb.wisc.edu/metabolomics/) or bulk data (http://www.bmrb.wisc.edu/ftp/pub/bmrb/metabolomics/standards_tar/). Besides the four main repositories, there are many derivative repositories, which are useful for evaluation of experimental NMR data such as LACS validation reports on assigned chemical shifts (http://www.bmrb.wisc.edu/ftp/pub/bmrb/validation_reports/LACS/) [8] and PACSY structural annotation server (http://pacsy.nmrfam.wisc.edu) [9]. As

many data repositories derived from the conventional NMR-STAR files have existed in different locations and formats, additional data handling processes have been required to obtain a full advantage of the BMRB archival data. Therefore, we have extended the NMR-STAR Dictionary (~/schema/mmcif_nmr-star.dic) to integrate other data repositories on BMRB such as the LACS validation reports, the PACSY structural annotation, other structural annotation for NMR structures by means of Protein Blocks [10] and information about completeness of assigned chemical shifts (Figure S3), the latter two data repositories have been generated by PDBj-BMRB group. Finally, the extended NMR-STAR Dictionary, reference ontology of the BMRB/XML Schema, defines total 421 categories and 5223 data items. Thus, the BMRB/XML that we have reported here would be the most comprehensive NMR-STAR data repository as a single format.

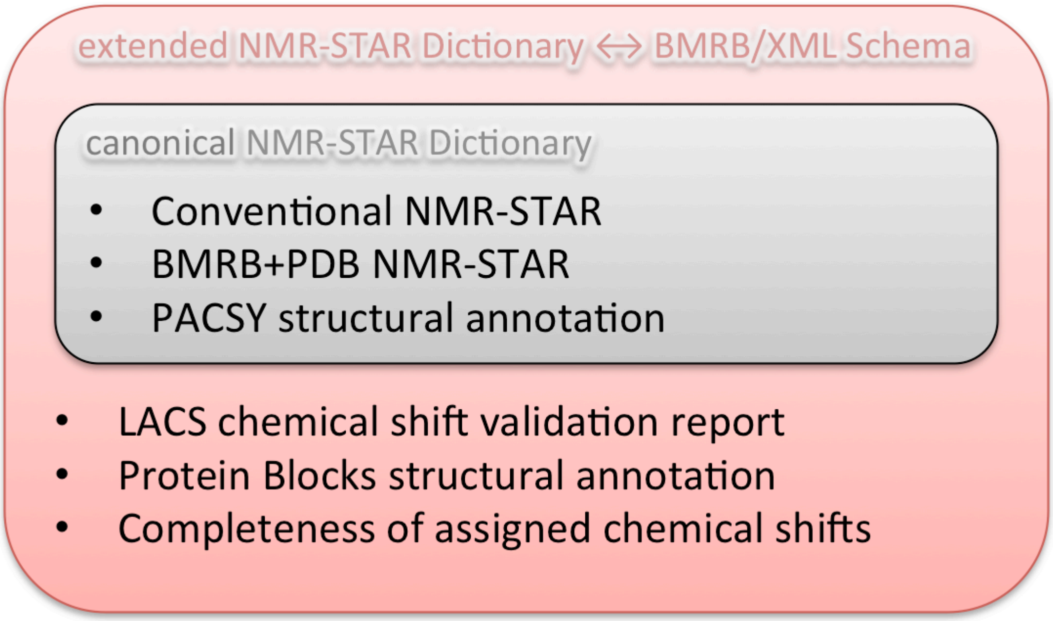

Figure S3. An Euler diagram showing that extended NMR-STAR Dictionary consists of canonical NMR-STAR Dictionary and extra definitions about related data repositories. It also shows relationship between the extended NMR-STAR Dictionary and the BMRB/XML Schema, having one-to-one correspondence.

1.3 BMRB/XML data files

In this study, we have archived to integrate both the original BMRB database reinforced by the data integration as described in the previous section and the Metabolomics database into collections of XML documents. The BMRB database has grown at a pace of approximately 800 entries per year and the number of released entries reached 10,446 as of October 16 2015. The two versions of BMRB/XML data files are available from ~/archive/xml/ for *complete* version, ~/archive/xml-noatom/ for *noatom* version, respectively. The former one contains the complete information content, whereas the latter one is a reduced version created by omitting bulky atomic coordinates, NMR restraints and peak lists used in the structure determination. For the BMRB database, BMRB/XML data files occupied 692 MB (*complete* version) and 626 MB (*noatom* version) after *gzip* compression. Those of the Metabolomics database, 1689 entries in total, occupied 20 MB (*complete*) and 18 MB (*noatom*), respectively.

1.4 XML schema validation and data remediation

BMRBxTool (~/download.html) is a software suit for the XML conversion and the XML schema validation, which is carried out with Apache Xerces (http://xerces.apache.org/) enabling full schema grammar constraint checking. Thus, the XML documents compliant with the standard [5]. Additionally, the BMRBxTool allows data correction during the XML conversion for the following potential errors:

(i) null data for mandatory entry fields, (ii) violations in enumerations, (iii) inconsistency of database accession codes, and (iv) typographical errors.

1.4.1 Null data for mandatory entry fields

Mandatory entry fields often represent parent-child relationships (aka foreign keys) between data items in the dictionary. For example, *entry.id* is principal parent data item of all categories in an entry. All parent data items correspond to XML *key* elements, and their associated children correspond to XML *keyref* elements in the XML Schema (Table S1). These relationships form the basis of the relational data model. Therefore, all XML *key* and *keyref* elements are defined as the mandatory entry field. Comparing with essential parent data items such as *entry.id*, *comp.id*, *chem_shift_list.id*, etc., values of minor parent data items have often been blank, then we tried to remediate null data for the mandatory entry fields by the following rules while preserving the original contexts.

1. When blanked XML *key* element appears once in an entry, then set value ‘1’ for both XML *key* and *keyref* elements.

2. Blanked XML *key* elements shown in Table S4 should be assigned incremental ID numbers by the order of appearances in the corresponding NMR-STAR data file.

3. When XML *key* elements shown in Table S4 appear more than once in an entry and if each XML *keyref* elements can be associated with XML *key* elements by peripheral data, then set the same value for a paired XML *keyref* and XML *key* elements.

4. Otherwise, fill ‘0’ as meaningless identifier.

Table S4. List of foreign keys that have been filled with incremental ID numbers by the order of appearances in the case of blank

Category	Item sorted by
entity_experiment_src	*id*
experiment	*id*
nmr_spectral_view	*id*
spectral_dim	*id*
study	*id*
entity_deleted_atom	*entity_atom_list_id*
entity_purity	*entity_id*
release	*release_number*

1.4.2 Violations in enumerators

We have manually curated violated enumerations. Almost all of modified enumerators have been already merged into the current NMR-STAR v3.1.1.65 Dictionary. Profiles for data regulation of total 114 enumerators are located in ‘schema’ subdirectory of the BMRBxTool. These data corrections are necessary for passing the XML schema validation.

1.4.3 Remediation of database accession codes

The inconsistencies in all entries of BMRB that have been found in the accession codes for the following databases were remediated; NCBI Taxonomy (http://www.ncbi.nlm.nih.gov/taxonomy/), NCBI PubMed

(http://www.ncbi.nlm.nih.gov/pubmed/), PDB/RDF (http://rdf.wwpdb.org/pdb/), chem_comp/RDF (http://rdf.wwpdb.org/cc/) and BMRB Internal Chemical Compound Library (used in the validation, annotation, and construction of BMRB entries by BMRB annotators). This task have utilized the data complement for the following categories; *entity_natural_src*, *entity_experimental_src*, *citation* and *chem_comp*. In particular, new 2,658 PubMed IDs and 9,149 DOIs in the *citation* category have been added in the BMRB/XML to the existing 7,409 PubMed IDs and 171 DOIs in the original NMR-STAR data.

We have also adjusted relations between database accession codes and database names, applying regular expression matching for the following data items:

assembly_db_link.accession_code,
assembly.enzyme_commission_number,
entity_db_link.accession_code,
entity.ec_number,
related_entries.database_accession_code

where we referred to the next sites about the regular expressions of various database accession codes:

http://web.expasy.org/docs/userman.html
http://www.ncbi.nlm.nih.gov/Sequin/acc.html
http://www.ncbi.nlm.nih.gov/books/NBK21091/

1.4.4 Statistics of data remediation by BMRBxTool

The null data for the mandatory entry fields was the first reason for redundant data remediation. There are many data items violating enumerators which are classified in the next three category groups, *chem_comp**, *order_param** and *struct_anno**. By correcting those trivial but improper data, we have cleaned the whole NMR-STAR data (Table S5), resulting that the most entries are logically consistent with corresponding enumerators defined by the XML Schema. Finally, we have obtained fully validated XML data collections for the both BMRB and Metabolomics databases.

Table S5. Statistics on data corrections in the BMRB entries during the XML conversion

Number of data corrected	Entries (Fraction %)	
0	5	(0.0)
1-5	4704	(45.0)
6-10	4332	(41.4)
11-20	1130	(10.8)
21-50	257	(2.4)
51-	18	(0.1)

2. BMRB/RDF

2.1 BMRB/OWL

2.1.1 Translation protocol from XML Schema to OWL ontology

The ontology of BMRB/RDF (BMRB/OWL, ~/schema/mmcif_nmr-star.owl) inherits basic scheme from PDBx ontology (http://rdf.wwpdb.org/schema/pdbx-v40.owl) based on the similarity of the two dictionaries [11]. The BMRB/OWL is generated from the BMRB/XML Schema. All metadata of XML Schema, except for a distinction between the primary keys and regular data items, were translated in RDF/RDFS/OWL syntax [12-14] (Table S6). Hierarchal structure in the XML Schema is reconstructed by using newly defined abstract OWL classes and RDF properties, of which labels are compatible with the PDBx ontology, such as category holders, category elements, cross-references, category items, etc. These abstract classes and properties exist only for compatibility of semantic architectures between the XML tree and the RDF directed graph, so end-users of the BMRB/RDF need to pay attentions on basic metadata in the NMR-STAR Dictionary, but not on ones in the BMRB/OWL. This can be preferable to both the semantic reasoner and the feed aggregators. Conversely, the expression of basic datatype properties, which may act as the categories and data items, is short and simple. For example, a category (category element in OWL) is simply expressed as BMRBo:*category_name* and a data item (category item in OWL) is expressed as a concatenated name consisting of the category and data item, namely,

BMRBo:*category_name.item_name*. These naming rules help users to comprehend document structure with higher similarity to the STAR syntax such as *category_name.item_name*.

As implemented in the BMRB/XML Schema file, we have embedded links to the NMR-STAR Dictionary reference service in BMRB/OWL file using rdfs:seeAlso property. Moreover, we have associated particular datatype properties as described in the PDBx OWL by using owl:equivalentProperty that provides not only the semantic reasoners with supplemental axioms, but items used in data exchange between members of the Worldwide PDB [15]. A list of pair of the equivalent datatype properties between BMRB/OWL and PDBx OWL is accessible at ~/schema/bmrb_pdbx_owl_equivalent_properties.csv. We have implemented the translation protocol including definitions of the abstract OWL classes and RDF properties, the STAR-compliant naming rules for the basic datatype properties and embedded links between different ontologies on a XSLT [16] code 'bmrbx2owl.xsl' bundled with BMRBoTool (~/download.html).

Table S6. Summary of correspondences between metadata of NMR-STAR Dictionary, BMRB/XML Schema, BMRB/RDF and BMRB/OWL

NMR-STAR Dic.[a]	BMRB/XML Schema[b]	Data type of BMRB/RDF[c]	Ontology elements of BMRB/OWL written in XPath syntax [7][d]
mmcif_nmr-star.dic	xmlns:BMRBx	xmlns:BMRBo	owl:Ontology[@rdf:about='http://bmrbpub.protein.osaka-u.ac.jp/schema/mmcif_nmr-star.owl#']
Datablock	*complexType*	datablock	owl:Class[@rdf:ID='datablock']
Datablock name	*attribute* of datablock	datablockName	owl:DatatypeProperty[@rdf:ID='datablockName']
Datablock-categories	*element* of datablock	has_*category_name*Category	/Datablock/rdfs:subClassOf/owl:Class/owl:intersectionOf[@rdf:parseType='Collection']/owl:Restriction/ owl:onProperty[@rdf:resource='#has_*category_name*Category']
(Datablock-categories)		of_datablock	owl:ObjectProperty[@rdf:ID='of_datablock']/[rdfs:domain[@rdf:resource='#CategoryElement'] and rdfs:range[@rdf:resource='#datablock']]
Parent-child	*key* of category	referenced_by_*keyref_name*[f]	owl:ObjectProperty[@rdf:ID='referenced_by_*category_name*']/[rdfs:subPropertyOf[@rdf:resource='#referenced_by'] and rdfs:range[@rdf:resource='#*category_name*']]
Parent-child	*keyref* of category	reference_to_*key_name*[f]	owl:ObjectProperty[@rdf:ID='referenced_to_*category_name*']/[rdfs:subPropertyOf[@rdf:resource='#referenced_to'] and rdfs:domain[@rdf:resource='#*category_name*']]
(Category holders in OWL[e])		*category_name*Category	owl:Class[@rdf:ID='*category_name*Category']/rdfs:subClassOf/owl:Class/owl:intersectionOf[@rdf:parseType='Collection']/owl:Class[@rdf:about='#Category']/owl:restriction/[owl:onProperty[@rdf:resource='#has_*category_name*'] and owl:minCardinality[@rdf:datatype='xsd:nonNegativeInteger']]
Category, (Category elements in OWL[e])	*complexType*	*category_name*	owl:Class[@rdf:ID='*category_name*']/rdfs:subClassOf/owl:Class/owl:intersectionOf[@rdf:parseType='Collection']/owl:Class[@rdf:about='#CategoryElement']/owl:restriction/owl:onProperty[@rdf:resource='#*category_name.item_name*']
Description	*annotation* of category		/Category_element/rdfs:comment/text()
Primary keys	*attribute* of category	*category_name.item_name*	owl:DatatypeProperty[@rdf:ID='*category_name.item_name*']/rdfs:subPropertyOf[@*category_name*Item]

Items	*element* of category	*category_name.item_name*	owl:DatatypeProperty[@rdf:ID='*category_name.item_name*']/rdfs:subPropertyOf[@*category_name*Item]
Description	*annotation* of item		/Item/rdfs:comment/text()
Mandatory code	*minOccurs* attribute of item		/Category_element/rdfs:subClassOf/owl:Class/owl:intersectionOf[@rdf:parseType='Collection']/owl:Class [@rdf:about='#CategoryElement']/owl:restriction/[owl:onProperty[@rdf:resource='#*category_name.item_name*'] and owl:minCardinality[@rdf:datatype='xsd:nonNegativeInteger']]
Data types	*string/integer/decimal*		/Item/rdfs:range[@rdf:resource='xsd:string' or @rdf:resource='xsd:integer' or @rdf:resource='xsd:decimal']
Enumeration	*restriction*		/Item/rdfs:range/owl:DataRange/owl:oneOf/rdf:List/rdf:first[@rdf:datatype='xsd:*data_type*' and .='*enum_value*']/rdf:rest/rdf:List/*
(Abstract class for generic category holders)			owl:Class[@rdf:ID='Category']
(Abstract class for generic category elements)			owl:Class[@rdf:ID='CategoryElement']
(Abstract datatype property for generic category items)			owl:DatatypeProperty[@rdf:ID='categoryItem']/rdfs:domain[@rdf:resource='#CategoryElement']
(Abstract property for generic cross-reference)			owl:ObjectProperty[@rdf:ID='crossReference']
(Abstract property for cross-reference between category elements: from child to parent)			owl:ObjectProperty[@rdf:ID='reference_to']/[rdfs:subPropertyOf[@rdf:resource='#crossReference'] and rdfs:domain[@rdf:resource='#CategoryElement'] and rdfs:range[@rdf:resource='#CategoryElement']]
(Abstract property for cross-reference between category elements: from parent to child)			owl:ObjectProperty[@rdf:ID='referenced_by']/[rdfs:subPropertyOf[@rdf:resource='#crossReference'] and rdfs:domain[@rdf:resource='#CategoryElement'] and rdfs:range[@rdf:resource='#CategoryElement']]
(Abstract property for generic category holders)			owl:InverseFunctionalProperty[@rdf:ID='hasCategory']/rdfs:domain[@rdf:resource='datablock']
(Abstract property for generic category elements)			owl:InverseFunctionalProperty[@rdf:ID='hasCategoryElement']
(Abstract property for genetic category items)			owl:DatatypeProperty[@rdf:ID='*category_name*Item']/[rdfs:subPropertyOf[@rdf:resource='#categoryItem'] and rdfs:domain[@rdf:resource='#*category_name*']]

[a]The values in parenthesis indicate undefined concepts for the NMR-STAR Dictionary.

[b]The prefix ‘xsd:’ for the metadata in italic font is omitted.

[c]The prefix ‘*BMRBo*:’ for the metadata without any prefix is omitted. Note that the PDBx ontology uses a prefix ‘*PDBo*:’ instead.

[d]The prefix ‘/rdf:RDF/’ for metadata written in XPath syntax is omitted. ‘/Datablock’, ‘/Category_element’ and ‘/Item’ indicate absolute location paths to metadata of the corresponding dictionary; datablock, category and data item, respectively. The cyan colored ontology elements highlight an important part of the context. The strings in italic font represent the symbols used in the metadata as nouns.

[e]The concept of a category in the NMR-STAR Dictionary is divided into two OWL classes, a category holder and category elements, in the BMRB/OWL.

[f]The parent-child relationships in the dictionary (*key* and *keyref* in the XML Schema) have been mapped to relations between two category elements (OWL classes) in the BMRB/OWL for the convenience of data exploring seen in Figure S11.

2.1.2 Comparison with other translation tool

It is noted that ReDeFer project (http://rhizomik.net/html/redefer/) has already released a suite package including a tool for translation of XML Schema to OWL ontology (XSD2OWL) and a tool for translation of XML to RDF based on the XSD2OWL (XML2RDF). The XSD2OWL is useful if all schema objects are identified by their name. However it doesn't support hierarchically separated named schema objects, which enable to identify an object by a data item and a particular category individually. For instance, the translation tool tries to associate a global 'id' datatype property with all data items: *entry.id*, *citation.id*, *atom_chem_shift.id*, etc. Therefore, the ReDeFer package could be applied to generation of neither BMRB/OWL nor BMRB/RDF.

2.2 BMRB/RDF data files

2.2.1 Translation protocol compliant with principles of Linked Data

The BMRB/RDF, generated from the *noatom* version of the BMRB/XML by XSL transformation, has been archived at ~/archive/rdf/. We have developed a XSLT code (~/schema/bmrbx2rdf.xsl.gz, bundled with the BMRBoTool), which supports the translation protocol described in development of the BMRB/OWL and realizes semantic interoperability in accordance with principles of Linked Data [17] and guidelines about Uniform Resource Identifier (URI) scheme widely accepted by biological database community. The procedure involves concurrent use of polite URIs to original information resource and persistent URIs provided by Identifier.org [18].

As for URI of ourselves, we have selected the following URI schemes: ‘info:bmrb/[0-9]+’ for conventional BMRB entries and ‘info:bmrb.metabolomics/bms[et][0-9]{6}’ for BMRB Metabolomics entries.

The NMR-STAR data have been already linked by allocating own syntax to various databases; such as PDB, PDB/Chemical Component Dictionary (aka. Chem comp), PDB/Ligand Expo, PubChem (https://pubchem.ncbi.nlm.nih.gov/), DOI (Digital Object Identifier), PubMed, ISSN (International Standard Serial Number), ISBN (International Standard Book Number), NCBI Taxonomy, Enzyme commission number, SCOP (http://scop.mrc-lmb.cam.ac.uk/scop/), UniProt (http://www.uniprot.org/), DDBJ, EMBL, GenBank, PIR, PRF, NCBI RefSeq (http://www.ncbi.nlm.nih.gov/refseq/) and BMRB itself. For example, the value of an *entity_natural_src.ncbi_taxonomy_id* data item has to refer to the NCBI Taxonomy ID, and has a value of ‘9606’. This case indicates that a source organism for a molecular entity is ‘Homo sapiens’. In order to comply with the fourth principles of Linked Data, the semantically equivalent resource is to be represented by a URI: http://purl.uniprot.org/taxonomy/9606. It is also no wonder Uniform Resource Names (URNs), urn:miriam:taxomony:9606, suit for persistence resource identifiers over the HTTP URLs [18]. RDF resources corresponding with values of the following data items defined in the NMR-STAR Dictionary can be associated with other database’s accession IDs expressed by URIs and URNs:

citation.doi,

citation.pubmed_id,

citation.journal_issn,

citation.book_isbn,

entity_natural_src.ncbi_taxonomy_id,

entity_experimental_src.host_org_ncbi_taxonomy_id,

assembly.enzyme_commision_number,

assembly_subsystem.enzyme_commission_number,

entity.ec_number,

struct_classification.sunid,

entry.assigned_pdb_id,

conformer_family_coord_set.pdb_accession_code,

representative_conformer.pdb_accession_code,

structure_annotation.pdb_id,

pb_list.pdb_id,

chem_comp.pdb_code,

chem_comp.pubchem_code,

assembly_db_link.accession_code,

entity_db_link.accession_code,

related_entries.database_accession_code,

chem_comp_db_link.accession_code

We have also implemented mapping rules of both URIs and URNs for the databases mentioned above on the XSLT code complying with the Linked Data principles. Figure S7 shows a typical example how external resource has been linked.

```xml
<BMRBo:entity_db_link rdf:about="http://bmrbpub.protein.osaka-u.ac.jp/rdf/bmr11300/entity_db_link/NP_001008202,REF,1,11300">
   <BMRBo:of_datablock rdf:resource="http://bmrbpub.protein.osaka-u.ac.jp/rdf/bmr11300"/>
   <BMRBo:entity_db_link.accession_code>NP_001008202</BMRBo:entity_db_link.accession_code>
   <BMRBo:entity_db_link.database_code>REF</BMRBo:entity_db_link.database_code>
   <BMRBo:entity_db_link.entity_id>1</BMRBo:entity_db_link.entity_id>
   <BMRBo:entity_db_link.entry_id>11300</BMRBo:entity_db_link.entry_id>
   <rdfs:seeAlso rdf:resource="http://www.ncbi.nlm.nih.gov/protein/NP_001008202"
                 rdfs:label="info:refseq/NP_001008202"/>
   <rdfs:seeAlso rdf:resource="http://identifiers.org/refseq/NP_001008202"
                 rdfs:label="urn:miriam:refseq:NP_001008202"/>
   <BMRBo:entity_db_link.author_supplied>no</BMRBo:entity_db_link.author_supplied>
   <BMRBo:entity_db_link.entry_mol_name>cell division cycle 5-like protein [Xenopus (Silurana) tropicalis]</BMRBo:entity_db_link.entry_mol_name>
   <BMRBo:entity_db_link.ordinal>16</BMRBo:entity_db_link.ordinal>
   <BMRBo:entity_db_link.seq_homology_expectation_val>8.17E-32</BMRBo:entity_db_link.seq_homology_expectation_val>
   <BMRBo:entity_db_link.seq_identity>100.00</BMRBo:entity_db_link.seq_identity>
   <BMRBo:entity_db_link.seq_positive>100.00</BMRBo:entity_db_link.seq_positive>
   <BMRBo:entity_db_link.seq_query_to_submitted_percent>82.86</BMRBo:entity_db_link.seq_query_to_submitted_percent>
   <BMRBo:entity_db_link.seq_subject_length>804</BMRBo:entity_db_link.seq_subject_length>
</BMRBo:entity_db_link>
```

Figure S7. An example of Linked Data implementation, where entity 1 of BMRB entry 11300 is linked to NCBI RefSeq NP_001008202 by using rdfs:seeAlso property. Two statements using rdfs:seeAlso appear, the former one represents the polite URL pointing original resource of NCBI RefSeq database and the resource has a label written in the formal URN, the latter one is a statement semantically equivalent to the former one, but utilizes a persistent URI resolving system of Identifiers.org with the MIRIAM URN [18].

2.2.2 Statistics on BMRB/RDF

The BMRB/RDF for the BMRB database consists of 560 M triples and has a file size of 1.1 GB after *gzip* compression. The Metabolomics database consists of 6 M triples and has a file size of 28 MB. Both BMRB/XML and BMRB/RDF data files follow the same logical body as their NMR-STAR data file counterparts. A typical example of a BMRB entry in NMR-STAR, XML, and RDF formats is shown in Figure S8.

A schematic RDF graph of linked databases is illustrated in Figure S9. The total number of RDFs linked to external information resources is 502,354. The top 58% of the RDF links connect BMRB with PDB. Subsequently, 13% of the links connect BMRB with nucleotide sequence database, DDBJ-EMBL-GenBank, and then 8% of the links are targeted to BMRB itself, indicating related BMRB entries (Table S10).

A


```
#############################################
#  Molecular system (assembly) description  #
#############################################

save_assembly
   _Assembly.Sf_category                      assembly
   _Assembly.Sf_framecode                     assembly
   _Assembly.Entry_ID                         15400
   _Assembly.ID                               1
   _Assembly.Name                            'F153(FTR) cTnC'
   _Assembly.BMRB_code                        .
   _Assembly.Number_of_components             2
   _Assembly.Organic_ligands                  .
   _Assembly.Metal_ions                       .
   _Assembly.Non_standard_bonds               no
   _Assembly.Ambiguous_conformational_states  .
   _Assembly.Ambiguous_chem_comp_sites        .
   _Assembly.Molecules_in_chemical_exchange   .
   _Assembly.Paramagnetic                     no
   _Assembly.Thiol_state                      .
   _Assembly.Molecular_mass                   18500
   _Assembly.Enzyme_commission_number         .
   _Assembly.Details                         'F153(FTR) cTnC'
   _Assembly.DB_query_date                    .
   _Assembly.DB_query_revised_last_date       .
```


B


```
<?xml version="1.0" encoding="UTF-8"?>
<BMRBx:datablock datablockName="15400"
  xmlns:BMRBx="http://bmrbpub.protein.osaka-u.ac.jp/schema/mmcif_nmr-star.xsd"
  xmlns:xsi="http://www.w3.org/2001/XMLSchema-instance"
  xsi:schemaLocation="http://bmrbpub.protein.osaka-u.ac.jp/schema/mmcif_nmr-star.xsd mmcif_nmr-star.xsd">
  <BMRBx:assemblyCategory>
    <BMRBx:assembly entry_id="15400" id="1">
      <BMRBx:details>F153(FTR) cTnC</BMRBx:details>
      <BMRBx:molecular_mass>18500</BMRBx:molecular_mass>
      <BMRBx:name>F153(FTR) cTnC</BMRBx:name>
      <BMRBx:non_standard_bonds>no</BMRBx:non_standard_bonds>
      <BMRBx:number_of_components>2</BMRBx:number_of_components>
      <BMRBx:paramagnetic>no</BMRBx:paramagnetic>
      <BMRBx:sf_category>assembly</BMRBx:sf_category>
      <BMRBx:sf_framecode>assembly</BMRBx:sf_framecode>
    </BMRBx:assembly>
  </BMRBx:assemblyCategory>
```


C


```
<?xml version="1.0" encoding="UTF-8"?>
<rdf:RDF xmlns:xsi="http://www.w3.org/2001/XMLSchema-instance"
         xmlns:rdfs="http://www.w3.org/2000/01/rdf-schema#"
         xmlns:rdf="http://www.w3.org/1999/02/22-rdf-syntax-ns#"
         xmlns:owl="http://www.w3.org/2002/07/owl#"
         xmlns:BMRBx="http://bmrbpub.protein.osaka-u.ac.jp/schema/mmcif_nmr-star.xsd"
         xmlns:BMRBo="http://bmrbpub.protein.osaka-u.ac.jp/schema/mmcif_nmr-star.owl#">
   <BMRBo:datablock rdf:about="http://bmrbpub.protein.osaka-u.ac.jp/rdf/bmr15400"
                    rdfs:label="info:bmrb/15400">
      <rdfs:seeAlso rdf:resource="http://bmrbpub.protein.osaka-u.ac.jp/xml/bmr/bmr15400-noatom.xml"/>
      <rdfs:seeAlso rdf:resource="http://www.bmrb.wisc.edu/ftp/pub/bmrb/entry_lists/nmr-star3.1/bmr15400.str"/>
      <rdfs:seeAlso rdf:resource="http://bmrb.pdbj.org/ftp/pub/bmrb/entry_lists/nmr-star3.1/bmr15400.str"/>
      <rdfs:seeAlso rdf:resource="http://bmrb.cerm.unifi.it/ftp/pub/bmrb/entry_lists/nmr-star3.1/bmr15400.str"/>
      <BMRBo:datablockName>15400</BMRBo:datablockName>
      <BMRBo:has_assemblyCategory>
         <BMRBo:assemblyCategory rdf:about="http://bmrbpub.protein.osaka-u.ac.jp/rdf/bmr15400/assemblyCategory">
            <BMRBo:has_assembly>
               <BMRBo:assembly rdf:about="http://bmrbpub.protein.osaka-u.ac.jp/rdf/bmr15400/assembly/15400,1">
                  <BMRBo:of_datablock rdf:resource="http://bmrbpub.protein.osaka-u.ac.jp/rdf/bmr15400"/>
                  <BMRBo:reference_to_entry>
                     <rdf:Description rdf:about="http://bmrbpub.protein.osaka-u.ac.jp/rdf/bmr15400/entry/15400">
                        <BMRBo:referenced_by_assembly rdf:resource="http://bmrbpub.protein.osaka-u.ac.jp/rdf/bmr15400/assembly/15400,1"/>
                     </rdf:Description>
                  </BMRBo:reference_to_entry>
                  <BMRBo:assembly.entry_id>15400</BMRBo:assembly.entry_id>
                  <BMRBo:assembly.id>1</BMRBo:assembly.id>
                  <BMRBo:assembly.details>F153(FTR) cTnC</BMRBo:assembly.details>
                  <BMRBo:assembly.molecular_mass>18500</BMRBo:assembly.molecular_mass>
                  <BMRBo:assembly.name>F153(FTR) cTnC</BMRBo:assembly.name>
                  <BMRBo:assembly.non_standard_bonds>no</BMRBo:assembly.non_standard_bonds>
                  <BMRBo:assembly.number_of_components>2</BMRBo:assembly.number_of_components>
                  <BMRBo:assembly.paramagnetic>no</BMRBo:assembly.paramagnetic>
                  <BMRBo:assembly.sf_category>assembly</BMRBo:assembly.sf_category>
                  <BMRBo:assembly.sf_framecode>assembly</BMRBo:assembly.sf_framecode>
               </BMRBo:assembly>
            </BMRBo:has_assembly>
         </BMRBo:assemblyCategory>
      </BMRBo:has_assemblyCategory>
```


Figure S8. Examples of NMR-STAR, BMRB/XML, and BMRB/RDF data representations. (A) NMR-STAR data file example of *assembly* category describing the molecular system for BMRB entry 15400. (B) The corresponding example in a BMRB/XML data file. (C) The corresponding example in a BMRB/RDF data file.

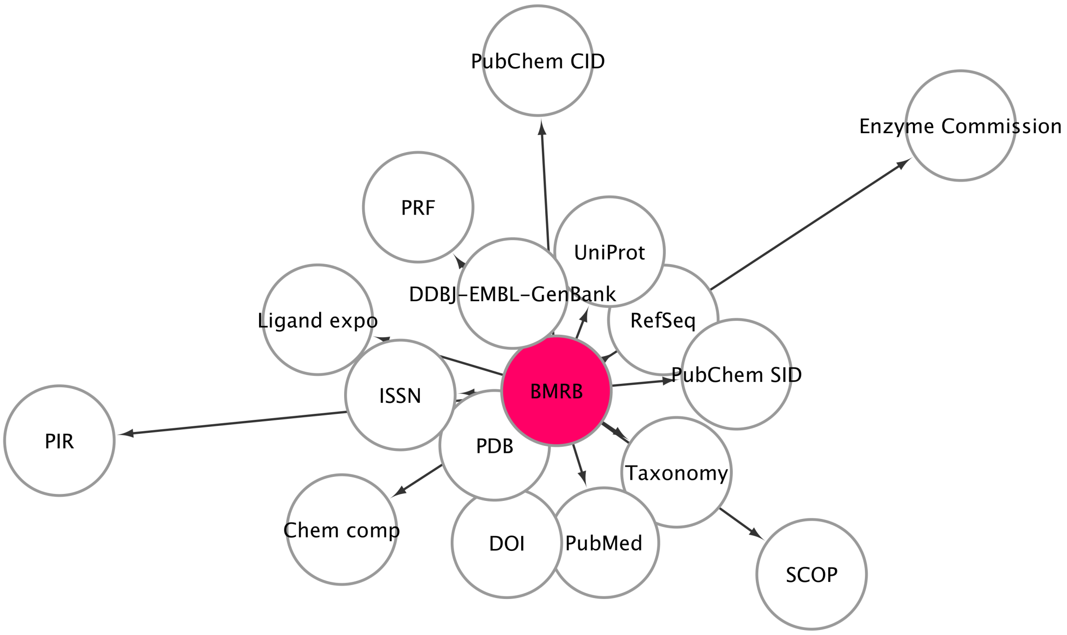

Figure S9. A schematic representation of linked external information resources, where shorter distances from BMRB represent closer relationships with BMRB. Cytoscape (http://www.cytoscape.org/) were used to generate this figure.

Table S10. Specifications of RDF links in BMRB/RDF[a]

Information resource	URI scheme	MIRIAM registry[d]	Resource	Link
PDB	info:pdb	pdb	RDF	289100
DDBJ-EMBL-Genbank	info:ddbj-embl-genbank[b]	ncbiprotein	HTML	67465
BMRB	info:bmrb	n/a	RDF	37815
NCBI RefSeq	info:refseq[b]	refseq	HTML	27004
NCBI Taxonomy	info:taxonomy	taxonomy	RDF(PURL[d])	15145
UniProt	info:uniprot	uniprot	RDF(PURL[d])	15041
PubMed	info:pmid[b]	pubmed	HTML	11288
ISSN	urn:ISSN[c]	issn	HTML	11000
DOI	info:doi[b]	doi	HTML	10542
PubChem SID	info:pubchem.substance	pubchem.substance	RDF	6436
PRF	info:prf	n/a	HTML	2957
PDB/Ligand Expo	info:pdb.ligand	pdb.ligand	HTML	2379
PDB/Chem comp	info:pdb-ccd	pdb-ccd	RDF	2352
SCOP	info:scop	scop	HTML	1335
PubChem CID	info:pubchem.compound	pubchem.compound	RDF	1247
Enzyme Commission	info:ec-code	ec-code	HTML	676
PIR	info:pir	n/a	HTML	566

[a]The reported statistics were obtained by adding RDF links for the both BMRB and Metabolomics databases, which were collected on October 16 2015. Sites linked less than 10 times are omitted.

[b]The "info" URI (Uniform Resource Identifier) schemes are formal registries maintained by NISO (National Information Standards Organization).

[c]The URN (Uniform Resource Name) scheme is registered URN scheme (RFC3040).

[d]The other "info" URI schemes without superscript annotation are provisionally defined and used in BMRB/RDF dataset to facilitate human readability of the information resource. The namespaces are compatible with the MIRIAM registries where prefix 'urn:miriam:' has been omitted.

[d]We preferred linking RDF resources written in PURLs (Persistent Uniform Resource Locators) rather than HTML ones, if available.

3. Data access

3.1 Basic look-up service

To expose the BMRB/RDF complying with the third principle of the Linked Data [17], users can look up any subject URIs, such as http://bmrbpub.protein.osaka-u.ac.jp/rdf/bmr15400, then the server returns information in machine-readable RDF/XML format, which is transformed immediately to a HTML document by embedded XSLT for human readability. It enables crawlers and people to explore the whole RDF graph through the unified web interface (Figure S11).

A

Result of the Query: http://bmrbpub.protein.osaka-u.ac.jp/rdf/bmr17000 (Subject)

Predicate	Object
rdf:type	BMRBo:datablock
rdfs:label	info:bmrb/17000
rdfs:seeAlso	http://bmrbpub.protein.osaka-u.ac.jp/xml/bmr/bmr17000-noatom.xml
rdfs:seeAlso	http://bmrb.cerm.unifi.it/ftp/pub/bmrb/entry_lists/nmr-star3.1/bmr17000.str
rdfs:seeAlso	http://bmrb.pdbj.org/ftp/pub/bmrb/entry_lists/nmr-star3.1/bmr17000.str
rdfs:seeAlso	http://www.bmrb.wisc.edu/ftp/pub/bmrb/entry_lists/nmr-star3.1/bmr17000.str
BMRBo:datablockName	17000
BMRBo:has_assemblyCategory	BMRBr:bmr17000/assemblyCategory
BMRBo:has_assigned_chem_shift_listCategory	BMRBr:bmr17000/assigned_chem_shift_listCategory
BMRBo:has_atom_chem_shiftCategory	BMRBr:bmr17000/atom_chem_shiftCategory
BMRBo:has_chem_shift_completeness_charCategory	BMRBr:bmr17000/chem_shift_completeness_charCategory
BMRBo:has_chem_shift_completeness_listCategory	BMRBr:bmr17000/chem_shift_completeness_listCategory
BMRBo:has_chem_shift_experimentCategory	BMRBr:bmr17000/chem_shift_experimentCategory
BMRBo:has_chem_shift_refCategory	BMRBr:bmr17000/chem_shift_refCategory
BMRBo:has_chem_shift_referenceCategory	BMRBr:bmr17000/chem_shift_referenceCategory

B

Result of the Query: http://bmrbpub.protein.osaka-u.ac.jp/rdf/bmr17000/atom_chem_shiftCategory (Subject)

Predicate	Object
rdf:type	BMRBo:atom_chem_shiftCategory
BMRBo:has_atom_chem_shift	BMRBr:bmr17000/atom_chem_shift/1,17000,1
BMRBo:has_atom_chem_shift	BMRBr:bmr17000/atom_chem_shift/1,17000,10
BMRBo:has_atom_chem_shift	BMRBr:bmr17000/atom_chem_shift/1,17000,100

C

Result of the Query: http://bmrbpub.protein.osaka-u.ac.jp/rdf/bmr17000/atom_chem_shift/1,17000,1 (Subject)

Predicate	Object
rdf:type	BMRBo:atom_chem_shift
BMRBo:atom_chem_shift.ambiguity_code	1
BMRBo:atom_chem_shift.assembly_atom_id	0
BMRBo:atom_chem_shift.assigned_chem_shift_list_id	1
BMRBo:atom_chem_shift.atom_id	H
BMRBo:atom_chem_shift.atom_isotope_number	1
BMRBo:atom_chem_shift.atom_type	H
BMRBo:atom_chem_shift.comp_id	MET
BMRBo:atom_chem_shift.comp_index_id	1
BMRBo:atom_chem_shift.entity_assembly_id	1
BMRBo:atom_chem_shift.entity_id	1
BMRBo:atom_chem_shift.entry_id	17000
BMRBo:atom_chem_shift.id	1
BMRBo:atom_chem_shift.seq_id	1
BMRBo:atom_chem_shift.val	8.522

Figure S11. Examples of look-up service for exploring the RDF graph. (**A**) A query result page for a BMRB entry 17000 (~/rdf/bmr17000) displays RDF triples representing datablock, labeled with URI 'info:bmrb/17000', and category holders. (**B**) A query result page for the *atom_chem_shift* category of the same entry (~/rdf/bmr17000/atom_chem_shiftCategory) displays a list of category elements. (**C**) A query result page for the *atom_chem_shift* category with an ID of 1 (~/rdf/bmr17000/atom_chem_shift/1,17000,1) displays an assigned chemical shift as category items.

3.2 SPARQL based query service (SPARQL endpoint)

A SPARQL based query service on the portal site (~/search/rdf/), implemented by OpenLink Virtuoso (http://virtuoso.openlinksw.com/) accepts SPARQL 1.1 queries [19]. Besides a friendly graphical interface, it allows users to submit a query file, which is preferable to develop flexible web applications. The next *curl* command posts a query file to the SPARQL endpoint.

```
curl -F “query=@FILE_PATH” http://bmrbpub.protein.osaka-u.ac.jp/search/rdf
```

In addition, we have prepared as many as thirty SPARQL query examples (~/exmples.html) to demonstrate how NMR experimental data can be retrieved and how to federate with other biological information resources. We present hereafter several remarkable results can be obtained by federating different types of databases.

3.3 Federated SPARQL query

The most important advantage of the SPARQL query is executing a query that joins remote SPARQL endpoints using the query variables in subqueries, which is often called federated SPARQL query [20].

3.3.1 Application to data exchange (Comparative survey of trends in publications between BMRB and PDB)

The following SPARQL query returns a list of MeSH (Medical Subject Headings, http://www.ncbi.nlm.nih.gov/mesh/) words in publications of a period of time, which

have PubMed IDs (*BMRBo:citation.pubmed_id*). We use a remote PubMed endpoint provided by Bio2RDF [21], to which <http://cu.pubmed.bio2rdf.org/sparql> in a 'SERVISE' clause of the query indicates. The obtained lists of various periods of time can reveal trends in the past biological NMR studies. We extracted newly appeared MeSH words in abstracts of publications cited from BMRB and PDB in the same period of time, respectively. Then, the obtained words were summarized in Figure S12 as a word cloud representation, where relative font sizes corresponded to quotation frequencies of the words to date. The word lists of PDB version were generated by use of a similar SPARQL query and an endpoint, in which we had stored the PDB/RDF. Total query execution time in the Virtuoso (Open-Source Edition 7.1) server implemented on a local PC (Intel Core i5 processer 3.4 GHz equipped with 32 GB RAM) was about 10 min.

SPARQL example 1.

```
PREFIX BMRBo: <http://bmrbpub.protein.osaka-u.ac.jp/schema/mmcif_nmr-star.owl#>
PREFIX pubmed_v: <http://bio2rdf.org/pubmed_vocabulary:>

SELECT ?name (COUNT(?name) AS ?count)                          # return values.
FROM <http://bmrbpub.protein.osaka-u.ac.jp/rdf/bmr>            # Graph URI of BMRB.
FROM <http://bio2rdf.org/pubmed>                               # Graph URI of PubMed.
WHERE {

  {

    SELECT DISTINCT ?pubmed_id ?name                           # subquery.
    WHERE {

      ?s_citation BMRBo:citation.pubmed_id ?pubmed_id ;
                  BMRBo:citation.year ?year .

      FILTER (bound(?pubmed_id) && xsd:integer(?year) >= 2001 && xsd:integer(?year) <= 2010)
       # Filtering by publication years.

      BIND (IRI(CONCAT("http://bio2rdf.org/pubmed:", ?pubmed_id)) AS ?s_pubmed)

      SERVICE <http://cu.pubmed.bio2rdf.org/sparql>
       # SPARQL endpoint for PubMed. If the server is down, please comment out a line above.
      {

        ?s_pubmed pubmed_v:mesh_heading ?s_meshhd .

        ?s_meshhd pubmed_v:mesh_descriptor_name ?mesh_descriptor .

      }

      FILTER NOT EXISTS {

        ?s_meshhd pubmed_v:mesh_qualifier_name ?mesh_qualifier .

      }

      BIND ((IF (CONTAINS(?mesh_descriptor, ","), STRBEFORE(?mesh_descriptor,
","), ?mesh_descriptor)) AS ?name_)
      BIND ((IF (CONTAINS(?name_, ","), STRBEFORE(?name_, ","), ?name_)) AS ?name)

      FILTER (?name NOT IN ("Magnetic Resonance Spectroscopy", "Nuclear Magnetic Resonance"))
      FILTER (?name NOT IN ("X-Ray Diffraction", "X-rays", "Crystallography",
"Crystallization"))
       # Filtering frequent obvious words.

    }

  }

} ORDER BY DESC(?count)
```

A. BMRB (1991~2000)

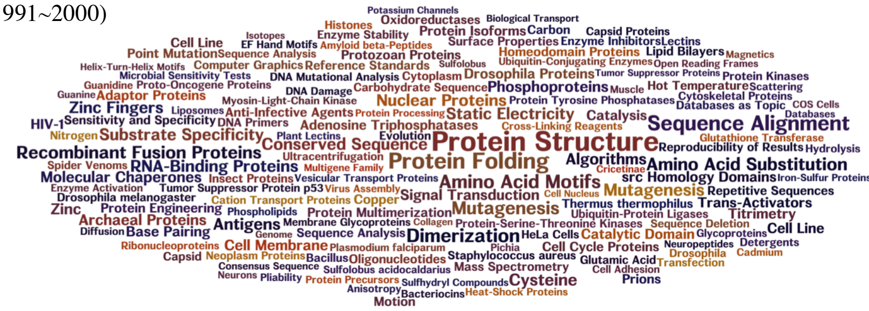

B. PDB (1991~2000)

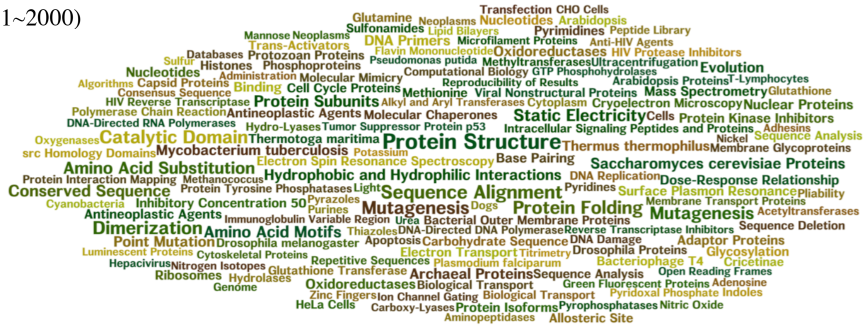

C. BMRB (2001~2010)

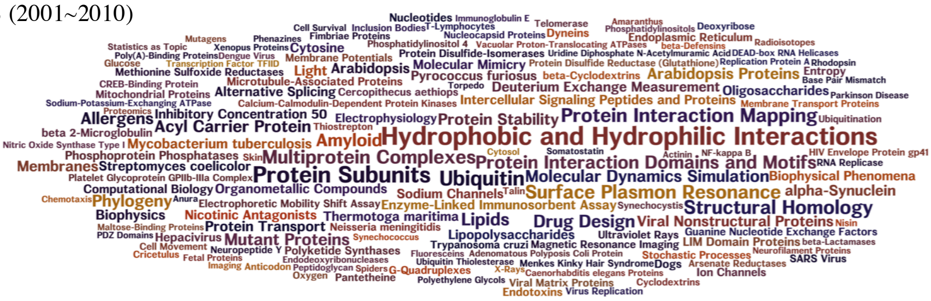

D. PDB (2001~2010)

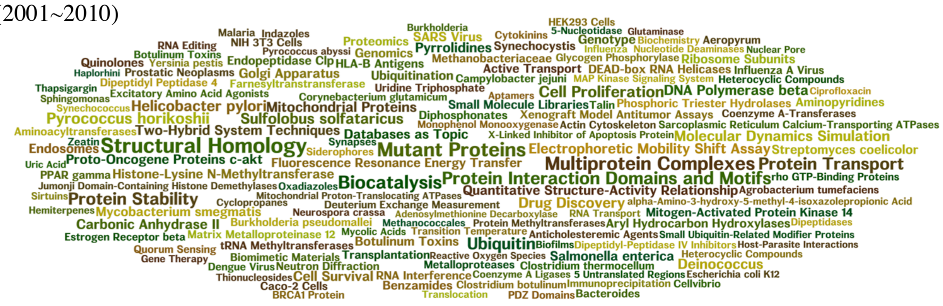

Figure S12. Word cloud representations of MeSH words derived from abstracts of publications cited from either BMRB or PDB. All figures were generated by Wordle service: http://www.wordle.net/. (**A**) Words in BMRB related publications from 1991 to 2000. (**B**) Words in PDB related publications from 1991 to 2000. (**C**) Words in BMRB related publications from 2001 to 2010. (**D**) Words in PDB related publications from 2001 to 2010.

The figures clearly suggest the similarities and differences of word trends between BMRB and PDB. Primary MeSH words in both BMRB and PDB resemble each other in all time periods. This is not surprising because almost all NMR structures have been archived in PDB occupying approximately 10% of total PDB entries. On the other hand, relative priorities of those common words and lesser-cited words make a contrast between NMR spectroscopy and X-ray crystallography even in the 1990's word cloud. For example, protein folding was the second major topic in BMRB, while global molecular structure and molecular evolution were key concepts in PDB. In the 2000's, the concerns of the two methodologies were distinguishable because more words reminiscent of molecular interaction increased in BMRB, conversely pathogen-relating words received much attention in PDB.

3.3.2 Application to knowledge discovery (Search and classification of SNPs in associated BMRB entities)

The next SPARQL query collects phenotypes annotated with the information for SNPs from the human genome in BMRB entities by integrating three SPARQL endpoints: BMRB, UniProt and OMIM (Online Mendelian Inheritance in Man, http://omim.org/), where the UniProt mediates between BMRB and OMIM. The SPARQL query code is surprisingly compact considering the quality of the information obtained.

SPARQL example 2.

```
PREFIX BMRBo: <http://bmrbpub.protein.osaka-u.ac.jp/schema/mmcif_nmr-star.owl#>
PREFIX rdfs: <http://www.w3.org/2000/01/rdf-schema#>
PREFIX omim_v: <http://bio2rdf.org/omim_vocabulary:>

SELECT DISTINCT ?entity_id ?uniprot_id ?label ?omim_id ?dbsnp_id ?mutation ?phenotype
      # return values.
FROM <http://bmrbpub.protein.osaka-u.ac.jp/rdf/bmr>
FROM <http://purl.uniprot.org/uniprot>                          # Graph URI of UniProt.
FROM <http://bio2rdf.org/omim>                                  # Graph URI of OMIM.
WHERE {

  ?s_up BMRBo:entity_db_link.entry_id ?entry_id ; # Please replace ?entry_id before you run.
        BMRBo:entity_db_link.entity_id ?entity_id ;
        BMRBo:entity_db_link.database_code "SP" ; # SP(SwissProt) represents UniProt.
        BMRBo:entity_db_link.accession_code ?uniprot_id ;
        rdfs:seeAlso ?s_uniprot .

  ?s_uniprot rdfs:label ?info .

  FILTER (STRSTARTS(?info, "info:uniprot"))

  SERVICE <http://sparql.uniprot.org/sparql>
      # SPARQL endpoint of UniProt.
  {

    ?s_uniprot rdfs:label ?label ;
               rdfs:seeAlso ?o_purl .

  }
  FILTER (STRSTARTS(STR(?o_purl), "http://purl.uniprot.org/mim/"))

  BIND (STRAFTER(STR(?o_purl), "http://purl.uniprot.org/mim/") AS ?omim_id)
  BIND (IRI(CONCAT("http://bio2rdf.org/omim:", ?omim_id)) AS ?s_omim)

  SERVICE <http://omim.bio2rdf.org/sparql>
      # SPARQL endpoint of OMIM (BIO2RDF).

  {

    ?s_omim omim_v:variant ?s_allele .

    ?s_allele omim_v:dbsnp ?s_dbsnp ;
              omim_v:mutation ?mutation ;
              rdfs:label ?phenotype .


    BIND (STRAFTER(STR(?s_dbsnp), "http://bio2rdf.org/dbsnp:") AS ?dbsnp_id)

  }

}
```

It is possible to correctly locate the residue numbers of the SNPs annotated by OMIM in the sequence of the associated BMRB entity by means of sequence alignment for the targeted UniProt entry using BLOSUM62. To automate these tasks, we wrote a Java program, which retrieves BMRB sequence using SPARQL (~/examples.html, see query number 5) and UniProt FASTA sequence file via Web API (http://www.uniprot.org/uniprot/######.fasta, where '#' is UniProt accession ID), followed by filtering if the coverage of the aligned sequence is more than 80%. Then,

we collected information of backbone chemical shifts and structural annotations related to the SNP related residues using SPARQL queries shown in the example page (see query number 22 and 24). The execution time for collecting SNPs of the associated BMRB entities was 125 min. (depending on how busy the remote endpoints were). Processing time for the consequent sequence alignments and collection of the backbone chemical shifts was 33 min. and the time for collection of the structural annotations was 50 min. on the PC as mentioned above. Finally, we found total 4597 SNPs in BMRB entities, 574 SNPs having backbone chemical shifts and 74 SNPs, with structural information. The obtained 74 residues were summarized with structural parameters archived in BMRB (Table S13). The query results suggest that the SNP relating residues are mainly found in hydrophobic environments, revealing large positive change in hydration free energy ($\Delta\Delta G_{hydr}$) and small solvent accessible surface areas (SASA). On the other hand, there is no tendency on types of protein secondary structure. This fact was confirmed by the prediction of secondary structure using the PSSI method [22] with the backbone chemical shifts for the 574 residues, in which there is no significant bias on distribution of the secondary structures; strand, coil and helix were 29.4%, 32.0% and 38.5%, respectively.

As the number of human proteins archived in BMRB is limited, it is not very easy to extract some statistical conclusions for the relationship of the experimentally determined structure and NMR data information to thus obtained information of SNP

phenotypes. Nevertheless from these query results, we can infer some biophysical effects on the targeted proteins which may cause by the mutations of genomic sequence; First, the terminations of polypeptide chain by introduction of stop codon in the DNA sequence obviously lead to the destruction of the native protein fold and its native functions (11 cases with ‘x’ code in a ‘Type’ column of the Table S13). Second, the mutation of inherently hydrophilic amino acids such as Arg, Lys and Pro, in a hydrophobic environment ($\Delta\Delta G_{hydr}$ > 7 kcal/mol, 10 cases coded by ‘y’) and mostly buried residues (rSASA < 10%, 14 cases coded by ‘b’) might significantly reduce the stability of the proteins. Third, the substitution of residues on the protein surface (rSASA > 50%) with different charge (13 cases coded by ‘c’) or bulky aromatic residues (4 cases coded by ‘a’) might affect protein-protein or protein-ligand interactions. (The other cases coded by ‘u’ have little relation with the structural parameters above.) The mutations coded by ‘u’ and ‘c’ may give rise to scientific interest because they disturb protein functions with a milder biophysical effect on the target proteins, which would be responsible for the phenotyping such as cell localization, molecular recognition and so on.

The original OMIM site provides further detailed information for all these SNPs using the OMIM IDs of Table S13. For example, destabilization and global unfolding in the M1775R mutated BRCT domain (OMIM: 113705) of BRCA1 (Breast Cancer susceptibility gene 1) have been reported [23]. For another example, OMIM: 300005,

resulting an A140V substitution in MBD domain of MECP2 (methyl CpG binding protein 2) can be found in a highly conserved region in an alpha helix lining on a wedge-shaped structure of the MBD domain that recognizes single symmetrically methylated CpG in the major groove of DNA [24-25].

Table S13. Phenotypes annotated SNPs having structural information in BMRB

Entry ID	Seq.	Res.	Mutation[a]	OMIM ID	dbSNP ID	DSSP[b]	rSASA[c] %	$\Delta\Delta G_{hydr}$[d] kcal/mol	Type[e]
4280	24	LEU	L100V	300005	rs28935168	C	22.5	3.9	u
4280	30	ARG	R106W	300005	rs28934907	E	8.0	11.8	y
4280	57	ARG	R133C	300005	rs28934904	C	49.4	7.2	y
4280	61	GLU	E137G	300005	rs61748392	H	41.7	6.6	u
4280	64	ALA	A140V	300005	rs28934908	H	42.0	1.7	u
4280	65	TYR	Y141X	300005	rs61748396	H	32.9	4.6	x
4280	79	PHE	F155S	300005	rs28934905	C	5.3	6.0	b
4280	82	THR	T158M	300005	rs28934906	T	26.7	5.1	u
4526	24	ARG	R24P	600160	rs104894097	C	53.6	6.8	c
4526	53	MET	M53I	600160	rs104894095	T	39.4	2.8	u
4526	56	SER	S56I	600160	rs104894109	T	24.3	4.8	u
4526	59	VAL	V59G	600160	rs104894099	H	0.1	5.8	b
4526	101	GLY	G101W	600160	rs104894094	C	58.7	-0.3	a
4526	114	PRO	P114S	600160	rs104894104	H	0.1	9.1	y
4526	126	VAL	V126D	600160	rs104894098	H	0.5	5.8	b
5177	35	ALA	A35T	601443	rs80358250	E	7.1	5.5	b
5224	26	ARG	R453W	150330	rs58932704	E	34.4	8.9	y
5224	38	GLY	G465D	150330	rs61282106	T	3.3	5.8	b
5224	44	ARG	R471C	150330	rs28928902	E	2.3	12.4	y
5224	55	ARG	R482W	150330	rs57920071	E	71.2	4.8	c/a
5224	55	ARG	R482L	150330	rs11575937	E	71.2	4.8	c
5224	55	ARG	R482Q	150330	rs11575937	E	71.2	4.8	c
5224	66	GLN	Q493X	150330	rs56699480	E	20.4	8.5	x
5224	100	ARG	R527P	150330	rs57520892	E	39.4	8.3	y
5224	100	ARG	R527H	150330	rs57520892	E	39.4	8.3	y
5224	100	ARG	R527C	150330	rs57318642	E	39.4	8.3	y
5224	102	ALA	A529V	150330	rs60580541	E	7.2	5.5	b
5224	103	LEU	L530P	150330	rs60934003	E	1.2	6.2	b
5224	115	LYS	K542N	150330	rs56673169	E	27.9	10.7	y
5363	14	ALA	A129V	602167	rs121909110	C	6.1	5.6	b
5482	74	ARG	R192W	300121	rs104894780	T	55.5	6.5	c/a
5534	41	TYR	Y63X	608083	rs120074116	H	78.3	-0.4	x
6093	27	ARG	R46G	107269	rs121909545	H	71.6	4.8	c
6114	22	MET	M1775R	113705	rs41293463	T	63.7	0.1	c
6114	100	TYR	Y1853X	113705	rs80357629	T	18.3	6.2	x
6384	41	CYS	C104R	604907	rs34557412	T	30.2	1.7	u
6579	51	GLY	G719S	131550	rs28929495	H	71.8	-1.7	u
6821	112	ILE	I113T	147450	rs74315452	T	1.6	5.8	b
10281	37	ARG	R240X	607108	rs121907917	H	17.1	10.8	x
10281	54	TRP	W257X	607108	rs121907929	H	6.5	7.3	x

10281	55	PHE	F258S	607108	rs121907925	H	0.1	6.6	b
10288	51	ARG	R276X	189907	rs121918672	H	52.1	6.9	x
10294	58	ARG	R89G	600037	rs104894464	H	40.6	8.2	y
11088	107	SER	S619W	602378	rs121909096	H	6.2	6.8	b
11088	107	SER	S619L	602378	rs121909095	H	6.2	6.8	b
11126	83	LEU	L253P	604985	rs121918306	C	51.9	0.7	u
11228	55	GLY	G55A	300429	rs121918361	E	3.6	5.8	b
15385	159	GLY	G159D	191040	rs104893823	C	67.9	-1.3	c
15388	159	GLY	G159D	191040	rs104893823	T	52.8	0.4	c
15591	8	PRO	P392L	601530	rs104893941	H	58.6	2.7	u
15592	8	PRO	P392L	601530	rs104893941	H	47.3	3.9	u
15693	31	ARG	R31H	167415	rs104893657	H	35.2	8.8	y
15693	40	GLN	Q40P	167415	rs104893656	H	62.6	3.9	u
15693	54	SER	S54G	167415	rs104893660	C	72.2	-0.5	u
15693	57	CYS	C57Y	167415	rs104893659	H	40.6	0.6	u
15693	62	LEU	L62R	167415	rs104893658	H	28.9	3.2	u
15693	108	ARG	R108X	167415	rs104893655	H	27.5	9.6	x
15996	40	CYS	C728X	217070	rs121964919	E	2.1	4.8	x
16119	64	LYS	K62X	607444	rs120074160	T	75.8	5.4	x
16386	5	GLU	E57K	604633	rs119489101	T	79.0	2.5	c
16485	81	PHE	F81L	609520	rs118204013	T	2.4	6.3	b
16590	23	ARG	R742X	173910	rs121918040	H	46.9	7.5	x
17243	70	GLY	G159D	191040	rs104893823	T	64.1	-0.9	c
17621	29	ARG	R742X	173910	rs121918040	H	32.3	9.1	x
17971	9	SER	S162F	602630	rs121909069	C	72.5	-0.5	a
18509	112	ILE	I113T	147450	rs74315452	T	18.9	3.9	u
18763	19	GLY	G375C	134934	rs75790268	H	55.3	0.1	u
18763	24	GLY	G380R	134934	rs28931614	H	2.1	5.9	b
18763	35	ALA	A391E	134934	rs28931615	H	19.5	4.1	u
19009	21	ALA	A692G	104760	rs63750671	C	52.3	0.5	u
19009	22	GLU	E693Q	104760	rs63750579	C	62.2	4.4	c
19009	22	GLU	E693G	104760	rs63751039	C	62.2	4.4	c
19009	22	GLU	E693K	104760	rs63750579	C	62.2	4.4	c
19009	34	LEU	L705V	104760	rs63750921	C	29.2	3.2	u

[a]The sequence numbers are aligned to the corresponding UniProt genes. The X codes represent termination of polypeptides.

[b]The DSSP codes (E: strand, C: coil, H: helix, T: turn) of the associated NMR structures were identified by STRIDE [26].

[c]Relative solvent accessible surface areas (rSASA) were calculated by the STRIDE. These processed data are accessible as values of

BMRBo:*struct_anno_char.secondary_structure_code* and *BMRBo*:*struct_anno_char.hydrophobicity, respectively.*

[d]Hydrophobicity scaled by hydration free energy ($\Delta\Delta G_{hydr}$) was estimated from linear correlation with the solvent accessible surface area (SASA) of each amino acid type [27].

[e]The codes of substitution types are defined as in the text.

3.3.3 Summary of SPARQL queries using BMRB/RDF

We showed two SPARQL query examples that applied to the data exchange and the knowledge discovery by integrating remote endpoints, which are a part of large RDF datasets collected on DataHub site (http://datahub.io/).

The SPARQL is versatile enough to manage those multiple RDF datasets. Besides, the API of the endpoint is so simple to mash-up not only RDF datasets but also non-RDF datasets. Thus, there is no limitation on availability of biological datasets in principle, whereas SPARQL implementation usually requires large computer resources for practical performance. Speculative execution for frequently used queries may be a key solution to improve the performance. Nevertheless, our results of the feasibility studies proved a promising prospect that the BMRB/RDF facilitates the data exchange between BMRB and other databases, and the implemented web services would encourage researchers to utilize the data archived in BMRB for their research on biological and life science problems by integration of enormous and diverse information resources. It must be emphasized that SPARQL endpoint providers such as Bio2RDF and BiMart [28] may play important roles for the federated search.

BioMart also provides a graphical interface for composing SPARQL queries and they succeed in providing unified data management platform for many different types of biological databases. In combination with our endpoint, it will be promising to sophisticate and potentiate the search for effective biomedical information of macromolecules with experimentally determined structural and NMR data.

REFERENCES

1. Ulrich EL, Akutsu H, Doreleijers JF, Harano Y, Ioannidis YE, Lin J, Livny M, Mading S, Maziuk D, Miller Z, Nakatani E, Schulte CF, Tolmie DE, Kent Wenger R, Yao H, Markley JL, (2008) **BioMagResBank.** *Nucleic Acids Res.*, 2008, 36, D402-D408.
2. Westbrook J, Bourne PE. **STAR/mmCIF: an extensive ontology for macro-molecular structure and beyond.** *Bioinformatics*, 2000, 16, 159-168.
3. Westbrook J, Henrick K, Ulrich EL, Berman HM. **The Protein Data Bank exchange dictionary. International Tables for Crystallography, G.** *Springer Netherlands*, 2005, 144-198. ISBN: 978-1-4020-4290-4.
4. **Extensible Markup Language (XML) 1.1 (Second Edition).** 2006, http://www.w3.org/TR/xml11/
5. **XML Schema Definition Language (XSD) 1.1 Part 1: Structures.** 2012, http://www.w3.org/TR/xmlschema11-1/
6. Westbrook J, Ito N, Nakamura H, Henrick K, Berman HM. **PDBML: the representation of archival macromolecular structure data in XML.** *Bioinformatics*, 2005, 21(7), 988-992.
7. **XML Path Language (XPath) Version 1.0.** 1998, http://www.w3.org/TR/xpath/
8. Wang L, Eghbalnia HR, Bahrami A, Markley JL. **Linear analysis of carbon-13 chemical shift differences and its application to the detection and correction of errors in referencing and spin system identifications.** *J. Biomol. NMR*, 2005, 32, 13-22.
9. Lee W, Yu W, Kim S, Chang I, Lee W, Markley JL. **PACSY, a relational database management system for protein structure and chemical shift analysis.** *J. Biomol. NMR*, 2012, 54, 169-179.
10. Joseph AP, Agarwal G, Mahajan S, Gelly JC, Swapna LS, Offmann B, Cadet F, Bornot A, Tyagi M, Valadié H, Schneider B, Etchebest C, Srinivasan N, De Brevern AG. **A short survey on protein blocks.** *Biophys. Review*, 2010, 2, 137-147.

11. Kinjo AR, Suzuki H, Yamashita R, Ikegawa Y, Kudou T, Igarashi R, Kengaku Y, Cho H, Standley DM, Nakagawa A, Nakamura H. **Protein Data Bank Japan (PDBj): maintaining a structural data archive and resource description framework format.** *Nucleic Acids Res.*, 2012, 40, D453-D460.
12. **RDF 1.1 Primer.** 2014, http://www.w3.org/TR/rdf11-primer/
13. **RDF Schema 1.1.** 2014, http://www.w3.org/TR/rdf-schema/
14. **OWL 2 Web Ontology Language Document Overview (Second Edition).** 2012, http://www.w3.org/TR/owl2-overview/
15. Berman HM, Kleywegt GJ, Nakamura H, Markley JL. **How Community Has Shaped the Protein Data Bank.** *Structure*, 2013, 21, 1485-1491.
16. **XSL Transformation (XSLT) Version 2.0.** 2007, http://www.w3.org/TR/xslt20/
17. Berners-Lee,T. **Linked Data, In Design Issues: Architectural and Philosophical Points.** 2006, http://www.w3.org/DesignIssues/LinkedData
18. Juty N, Le Novère N, Laibe C. **Identifiers.org and MIRIAM Registry: community resources to provide persistent identification.** *Nucleic Acids Res.*, 2012, 40, D580-D586.
19. **SPARQL 1.1 Query Language.** 2013, http://www.w3.org/TR/sparql11-query/
20. DuCharme B. **Learning SPARQL.** *O'Reilly Media, Inc.* 2011, ISBN: 978-1-449-30659-5.
21. Belleau F, Nolin MA, Tourigny N, Rigault P, Morissette J. **Bio2RDF: Towards a mashup to build bioinformatics knowledge systems.** *J. Biomed. Inform.*, 2008, 41, 706-716.
22. Wang Y, Jardetzky O. **Probability-based protein secondary structure identification using combined NMR chemical-shift data.** *Protein Science*, 2002, 11, 852–861.
23. Willams RS, Glober JN. **Structural consequences of a cancer-causing BRCA1-BRCT missense mutation.** *J. Biol. Chem.*, 2003, 278(4), 2630-2635.
24. Orrico A1, Lam C, Galli L, Dotti MT, Hayek G, Tong SF, Poon PM, Zappella M, Federico A, Sorrentino V. **MECP2 mutation in male patients with non-specific X-linked mental retardation.** *FEBS Lett.*, 2000, 481, 285-288.

25. Ho KL, McNae IW, Schmiedeberg L, Klose RJ, Bird AP, Walkinshaw MD. **MeCP2 binding to DNA depends upon hydration at methyl-CpG.** *Mol. Cell.*, 2008, 29, 525-531.
26. Heinig M, Frishman D. **STRIDE: a Web server for secondary structure assignment from known atomic coordinates of proteins.** *Nucleic. Acids Res.*, 2004, 32, W500-W502.
27. Samanta U, Bahadur RP, Chakrabarti P. **Quantifying the accessible surface area of protein residues in their local environment.** *Protein Eng.*, 2002, 15, 659-667.
28. Smedley D, Haider S, Durinck S, Pandini L, Provero P, Allen J, Arnaiz O, Awedh MH, Baldock R, Barbiera G, Bardou P, Beck T, Blake A, Bonierbale M, Brookes AJ, Bucci G, Buetti I, Burge S, Cabau C, Carlson JW, Chelala C, Chrysostomou C, Cittaro D, Collin O, Cordova R, Cutts RJ, Dassi E, Di Genova A, Djari A, Esposito A, Estrella H, Eyras E, Fernandez-Banet J, Forbes S, Free RC, Fujisawa T, Gadaleta E, Garcia-Manteiga JM, Goodstein D, Gray K, Guerra-Assunção JA, Haggarty B, Han DJ, Han BW, Harris T, Harshbarger J, Hastings RK, Hayes RD, Hoede C, Hu S, Hu ZL, Hutchins L, Kan Z, Kawaji H, Keliet A, Kerhornou A, Kim S, Kinsella R, Klopp C, Kong L, Lawson D, Lazarevic D, Lee JH, Letellier T, Li CY, Lio P, Liu CJ, Luo J, Maass A, Mariette J, Maurel T, Merella S, Mohamed AM, Moreews F, Nabihoudine I, Ndegwa N, Noirot C, Perez-Llamas C, Primig M, Quattrone A, Quesneville H, Rambaldi D, Reecy J, Riba M, Rosanoff S, Saddiq AA, Salas E, Sallou O, Shepherd R, Simon R, Sperling L, Spooner W, Staines DM, Steinbach D, Stone K, Stupka E, Teague JW, Dayem Ullah AZ, Wang J, Ware D, Wong-Erasmus M, Youens-Clark K, Zadissa A, Zhang SJ, Kasprzyk A. **The BioMart community portal: an innovative alternative to large, centralized data repositories**, *Nucleic Acids Res.*, 2015, 43, W589-W598.
